# Screening for Malnutrition, Sarcopenia, and Physical Frailty Beyond One Year after Liver Transplantation

**DOI:** 10.1016/j.jceh.2025.103421

**Published:** 2025-11-29

**Authors:** Amal Trigui, Crystèle Hogue, Mélanie Tremblay, Geneviève Huard, Christopher F. Rose, Chantal Bémeur

**Affiliations:** ∗ Department of Nutrition, Faculty of Medicine, Université de Montréal, Montreal, QC H3T 1A8, Canada; † Centre de Recherche du Centre Hospitalier de l’Université de Montréal (CRCHUM), Montreal, QC H2X 0A9, Canada; ‡ Liver Unit, CHUM, Montreal, QC H2X 3E4, Canada; § Department of Medicine, Faculty of Medicine, Université de Montréal, Montreal, QC H3T 1J4, Canada

**Keywords:** liver transplantation, malnutrition, sarcopenia, physical frailty, muscle function

## Abstract

**Background/Aims:**

Malnutrition, sarcopenia, and frailty negatively impact quality of life and increase mortality following liver transplantation (LT). However, long-term follow-up data remain limited. This study aimed primarily to assess the malnutrition risk at 1-, 2-, and 3-years post-LT. Secondary objectives included evaluating the sarcopenia and frailty risk, muscle function, physical activity, quality of life, and employment status at the same time points.

**Methods:**

This cross-sectional study included LT recipients with a history of cirrhosis, transplanted between January 2019 and December 2021. Each participant completed a single virtual meeting during which nutritional risk, sarcopenia, frailty risk, muscle function, physical activity, quality of life, employment status, and dietary intakes were assessed.

**Results:**

Sixty-six LT recipients (63.6% male) were included: cohort A (1-year post-LT, n = 25), cohort B (2 years post-LT, n = 14), and cohort C (3 years post-LT, n = 27). The prevalence of malnutrition (12.0%, 14.3%, and 11.1%), sarcopenia (16.0%, 28.6%, and 18.5%), and frailty risks (12.0%, 28.6%, and 18.5%) in cohorts A, B, and C, respectively, remained stable over time (*P* = 0.957, 0.626, and 0.436). Energy intake was a significant predictor of both malnutrition and sarcopenia, with muscle function predicting the risk of sarcopenia and frailty post-LT. Muscle function was lowest in cohort B and inferior in age-matched adults in all cohorts. One-third of patients had low physical activity levels, with no significant change across cohorts (*P* = 0.096). Quality of life remained unchanged, except for lower emotional well-being and health change scores in cohort C compared to cohort A (*P* = 0.039 and *P* < 0.001, respectively). Only 28.0%, 42.9%, and 25.9% of participants in cohorts A, B, and C, respectively, returned to work.

**Conclusion:**

Up to 3 years after LT, patients were still at risk of malnutrition, sarcopenia, and frailty. The results of this study highlight the need for targeted interventions to improve outcomes and support long-term quality of life post-LT.

Malnutrition is commonly observed in patients with cirrhosis with a prevalence ranging from 40% to 90% in patients awaiting liver transplantation (LT).^1^ Malnutrition is associated with more frequent hospitalizations and increased morbidity and mortality after LT.^2^ LT is expected to improve a patient's nutritional status as many metabolic alterations contributing to malnutrition in patients with cirrhosis are due to the liver's inability to regulate energy metabolism and maintain adequate protein synthesis.^3^ However, after LT, malnutrition often persists, and the nutritional status may even deteriorate.^3^ The financial burden of malnutrition (hospital costs) after LT is approximately 30% higher in severely malnourished patients compared to well-nourished recipients.^2^

Sarcopenia and frailty are two distinct clinical phenotypes of malnutrition.^4^ Sarcopenia is defined by the European Working Group on Sarcopenia in Older People (EWGSOP) as low muscle strength and low muscle mass or quality.^5^ In the context of liver disease, sarcopenia frequently refers to the loss of muscle mass,^6^ which is strongly associated with adverse outcomes and mortality post-LT.^7^ Frailty, on the other hand, is characterized by a loss in physical, physiological, psychological, and social functioning.^8^ In the context of liver disease, the term frailty mainly refers to physical frailty as a phenotypic manifestation of the loss of muscle function.^6^ It is strongly linked to mortality, risk of falls, depression, and reduced quality of life.^4^

Quality of life is increasingly recognized as a significant outcome following LT, reflecting an individual's perception of health, including physical, mental, and social well-being.^9^ In the first year following LT, recipients generally report a quality of life similar to that of the general population, although it may deteriorate after that period.^10^ Among factors that significantly impact quality of life, returning to work plays a crucial role in social reintegration as it not only provides a sense of purpose and identity but also helps restore a connection to the community.^11^

The link between malnutrition, sarcopenia, and frailty and a higher risk of complications and death after LT is well recognized.^12^ However, beyond the first year post-LT, there is a lack of data on malnutrition, sarcopenia, and frailty. This study primarily aimed to assess the prevalence of malnutrition risk in LT recipients at 1-, 2-, and 3-years post-transplant. Secondary objectives included assessing, at the same time points, the prevalence of sarcopenia and frailty risk, as well as characterizing muscle function, physical activity, quality of life, and employment status. Predictors of post-LT long-term risk of malnutrition, sarcopenia, and frailty were evaluated.

## METHODS

### Recruitment

This cross-sectional prospective study included patients who underwent LT at the *Centre Hospitalier de l'Université de Montréal* (CHUM), Montreal, Canada, between January 2019 and December 2021. The inclusion criteria were patients aged ≥18 years, who spoke French or English and who were transplanted due to cirrhosis. Cirrhosis was diagnosed using standard methods, such as medical history, biological, clinical, pathological, and/or radiological assessments and confirmed by biopsy of the explanted liver. The exclusion criterion was patients who had undergone multiple organ transplants. During 2022, a single, one-on-one virtual Zoom meeting was held with enrolled patients to administer questionnaires and conduct tests. The meeting lasted approximately 1 h. According to the year of LT, participants were divided into three groups: cohort A (2021, 1-year post-LT), cohort B (2020, 2-years post-LT), and cohort C (2019, 3-years post-LT). The study was conducted in accordance with the Declaration of Helsinki and approved by the CHUM Research Ethics Committee (#21–370). Written informed consent was obtained from all participants prior to enrollment.

### Demographics and Medical History

Demographic characteristics (age, sex, and ethnicity) and clinical history (time on the waiting list, length of hospital stay following LT, re-transplantation, etiology of liver disease, Child–Pugh score and model for end-stage liver disease sodium [MELD-Na] score at the time of LT) were collected from CHUM's medical records. Time since LT was calculated as the difference between the date of LT and the date of data collection for each participant. Medications known to affect muscle health were recorded, including antidiabetic medications, statins, immunosuppressive drugs, and vitamin D.^13^ The presence of the following comorbidities at the time of LT and at data collection was also noted: type 2 diabetes, new-onset diabetes after transplant (NODAT), hypertension, overweight (body mass index [BMI] = 25–30 kg/m^2^), obesity (BMI >30 kg/m^2^), dyslipidemia, metabolic syndrome, cardiovascular diseases, and chronic renal failure.

The metabolic syndrome diagnosis was made based on the National Cholesterol Education Program Adult Treatment Panel III.^14^ Metabolic syndrome was reported if three or more of the following five criteria were met: waist circumference over 102 cm (males) or 88 cm (females), blood pressure over 130/85 mmHg, fasting triglyceride level over 1.7 mmol/L, fasting high-density lipoprotein cholesterol level less than 1.04 mmol/L (males) or 1.29 mmol/L (females), and fasting blood sugar over 5.6 mmol/L. Since data were collected from medical records, waist circumference was not always available. For that reason, waist circumference was replaced with BMI according to the updated International Diabetes Federation definition, which states that if BMI is greater than 30 kg/m^2^, central obesity can be assumed, and waist circumference is not required.^15^

### Screening for Malnutrition Risk

The risk of malnutrition was assessed using the Canadian Nutritional Screening Tool (CNST), which has been validated and tested for reliability in Canadian hospitals,^16^ and is recommended by the Canadian Malnutrition Task Force for malnutrition screening.^17^ The CNST is a simple, two-item yes/no questionnaire assessing weight loss in the past 6 months and reduced food intake for more than a week. Two “YES” answers indicate a risk of developing malnutrition.

### Screening for Sarcopenia Risk

The risk of sarcopenia was assessed using the SARC-F questionnaire, which evaluates five domains: Strength (S), Assistance with walking (A), Rising from a chair (R), Climbing stairs (C), and Falls (F). This tool consists of five questions that assess the patient's self-reported limitations in these areas, each scored from 0 to 2, with a total score ranging from 0 to 10. A total score of 4 or higher is predictive of sarcopenia and associated with poor outcomes.^18^ Currently, the EWGSOP recommends the use of the SARC-F questionnaire for the rapid screening of sarcopenia.^5^

### Screening for Frailty Risk

The risk of frailty was assessed using the FRAIL scale, a validated questionnaire that evaluates five components: fatigue, resistance, ambulation, illnesses, and weight loss (more than 5%). The total score ranges from 0 to 5, with each of the five items presented as a yes/no question. Based on the score, individuals are classified as follows: no frailty (0), prefrailty,^1^^,^^2^ and frailty (≥3).^19^

### Muscle Function

Muscle function was assessed using the chair stand test (CST), which provides a functional evaluation of muscle strength and endurance.^20^ Starting from a seated position, participants were asked to stand up and sit down without using their arms (arms crossed over their chest) for a total of five times without assistance. The test was performed three times, and the average completion time was recorded. Patients unable to perform the test were assigned a score of 32 s.^20^ A completion time exceeding 15 s indicates reduced muscle function.^5^

### Physical Activity

Physical activity was assessed using the short version of the self-administered International Physical Activity Questionnaire (IPAQ), a widely used tool for evaluating physical activity.^21^ The IPAQ consists of 7 questions measuring time spent on vigorous (VA), moderate (MA), and walking (W) activities over the past week, as well as sedentary habits. The metabolic equivalent (MET) per week was calculated using the following formula: MET = 4 ∗ VA (days ∗ minutes) + 2 ∗ MA (days ∗ minutes) + 1/3 ∗ W (minutes).^21^ The IPAQ classifies physical activity into high, moderate, and low levels. The high activity level corresponds to individuals who perform VA activity on at least 3 days, accumulating ≥1500 MET-min/week, or a combination of activities on at least 7 days, reaching ≥3000 MET-min/week. A MA activity level corresponds to VA activity on ≥3 days (≥20 min/day), or MA activity or walking on ≥5 days (≥30 min/day), or a combination of activities on ≥5 days totaling ≥600 MET min/week. A low activity level indicates that the individual does not meet the criteria for MA or high activity levels.^22^

### Quality of Life

Quality of life was assessed using the Short Form 36 (SF-36) questionnaire, a validated and widely used tool for evaluating participants' perceptions of their quality of life.^23^, ^24^, ^25^ The SF-36 consists of 36 questions across eight health dimensions: physical functioning, role limitations due to physical health, role limitations due to emotional problems, social functioning, pain, energy/fatigue, emotional well-being, and general health. Responses are scored from 0 to 100, with each dimension comprising 2 to 10 questions. The domain scores are further grouped into overall physical and mental health scores. Additionally, the SF-36 includes a question regarding the evolution of health as perceived by the patient. Higher scores reflect better quality of life.^26^

### Employment Status and Work Capacity

Employment status and work capacity were assessed using five questions developed for LT recipients.^27^ These questions evaluate the patient's employment status following LT, time to return to work, work capacity, and daily functional ability compared to the week prior to surgery.^27^

### Dietary Assessment

Dietary intake was assessed using a web-based, self-administered 24 h dietary recall (R24W) developed at *Université Laval*.^28^, ^29^, ^30^ The web-based R24W has been previously validated and shown to have high relative validity and a low misclassification rate compared to standard reference methods in a cohort of French Canadians.^28^^,^^29^ Participants were invited by e-mail to complete the R24W on 2 unannounced days (1 weekend day and 1 weekday) randomly selected by a computer algorithm over a 7-day period.

### Statistical Analysis

Statistical analysis was performed using SPSS (version 29.0, IBM Inc.). Continuous data were assessed for normality with the Shapiro–Wilk test and presented as median (25th to 75th percentile). Categorical variables were presented as percentages. Patients’ characteristics, comorbidities, risk of malnutrition, sarcopenia, frailty, physical function, physical activity, quality of life, and oral medications in cohorts A, B, and C were compared using the Kruskal–Wallis test for continuous variables and the Chi–Square test for categorical variables. Following significant Kruskal–Wallis or Chi-square tests, post-hoc pairwise comparisons were performed to identify which groups differ significantly. A comparison of comorbidities at LT and at data collection was performed using the McNemar test. Univariate and multivariate logistic regressions were conducted for risk factors of malnutrition, sarcopenia, and frailty, including age, sex, time since LT, comorbidities, medications, immunosuppressive agents, physical function, physical activity, and nutritional intake. Collinearity was assessed using the variance inflation factor (VIF), with a threshold of 2. Variables with a *P*-value <0.05 in univariate analysis were included in the multivariate logistic regression using a forward-selection model based on the likelihood ratio. A *P*-value <0.05 was considered statistically significant for all other tests. The figures were created in https://BioRender.com.

## RESULTS

### Patients’ Characteristics

**Figure 1:**
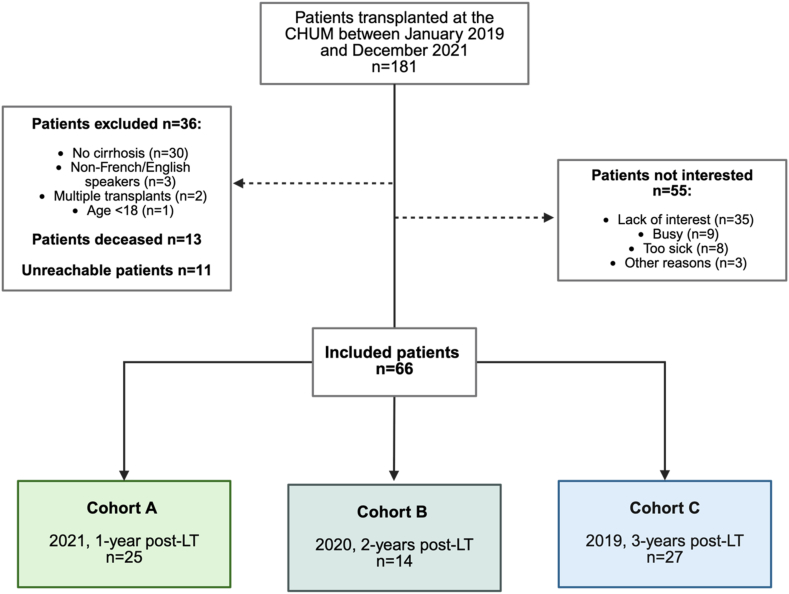
Flow chart. LT, liver transplantation.

A total of 66 patients were included in this study (cohort A, n = 25; cohort B, n = 14; and cohort C, n = 27) (Figure 1). Except for time since LT, no significant differences were found in patient characteristics between the three cohorts (*P* > 0.05) (Table 1). The median age at data collection was 63 years (53.8–65.3), with a male:female ratio of approximately 2:1 (63.6% male). Sixty-five participants were French-speaking, and one was English-speaking. The median hospital stay after LT was 27 days (19.8–40.3), and 4 patients underwent re-transplantation. The most common etiology of cirrhosis was nonalcoholic steatohepatitis (NASH) (34.8%), followed by alcoholic cirrhosis (33.3%). Concomitant hepatocellular carcinoma (CHC) was present in 34.8% of patients at LT; none of the patients had experienced cancer recurrence at time of data collection. At LT, 19.7% of patients were classified as Child–Pugh A, 30.3% as Child–Pugh B, and 50.0% as Child–Pugh C. Most Child–Pugh A patients had Hepatocellular Carcinoma (HCC) and received MELD exception points. The median MELD-Na at LT was 26^23^, ^24^, ^25^, ^26^, ^27^, ^28^, ^29^, ^30^ among patients without exceptions (n = 36). Of the remaining patients, 29 had MELD exception scores (mainly for HCC) and one had a 3F status with the highest priority (Table 1).

**Table 1:** Characteristics of Patients (n = 66).

	Total n = 66	Cohort A 1-year post-LT n = 25	Cohort B 2-years post-LT n = 14	Cohort C 3-years post-LT n = 27	*P*-value^c^
Age at data collection, years	63.0 (53.8–65.3)	60.0 (49.5–64.0)	63.0 (57.3–65.5)	64.0 (57.0–67.0)	0.159
Sex male/female, n (%)	42/24 (63.6/36.4)	18/7 (72.0/28.0)	10/4 (71.4/28.6)	14/13 (51.9/48.1)	0.254
Ethnicity, n (%)					0.601
Caucasian from North America	61 (92.4)	22 (88.0)	14 (100)	25 (92.6)	
Latin American	3 (4.5)	2 (8.0)	0 (0)	1 (3.7)	
European	1 (1.5)	0 (0)	0 (0)	1 (3.7)	
African	1 (1.5)	1 (4.0)	0 (0)	0 (0)	
Time since LT, days	**817.0 (449.8–1171.0)**	**429 (403.0**–**469.5)**	**813 (792.0–824.5)**	**1186 (1162.0–1214.0)**	<**0.001**
Time on the waiting list, days	95.5 (18.3–434.5)	100.0 (11.5–443.0)	145.0 (6.8–601.0)	84.0 (28.0–382.0)	0.826
Hospital length of stay after LT, days	27.0 (19.8–40.3)	26.0 (18.5–40.5)	27.0 (18.8–45.0)	27.0 (20.0–44.0)	0.934
Retransplantation, n (%)	4 (6.1)	1 (4.0)	1 (7.1)	2 (7.4)	0.860
Etiology of liver disease at LT, n (%)^a^					
NASH	23 (34.8)	6 (24.0)	7 (50.0)	10 (37.0)	0.251
Alcohol	22 (33.3)	11 (44.0)	3 (21.4)	8 (29.6)	0.310
Cholestatic (PBC/PSC)	18 (27.3)	8 (32.0)	5 (35.7)	5 (18.0)	0.401
Viral	13 (19.7)	4 (16.0)	2 (14.3)	7 (25.9)	0.566
Autoimmune	7 (10.6)	1 (4.0)	1 (7.1)	5 (18.5)	0.211
Other	10 (15.2)	6 (24.0)	2 (14.3)	2 (7.4)	0.248
HCC at LT, n (%)	23 (34.8)	9 (36.0)	4 (28.6)	10 (37.0)	0.854
Child–Pugh (A/B/C) at LT, n (%)	13/20/33 (19.7/30.3/50.0)	6/6/13 (24.0/24.0/52.0)	1/7/6 (7.1/50.0/42.9)	6/7/14 (22.2/25.9/51.9)	0.418
MELD-Na score at LT (n = 36)	26 (23–30)	27 (23–31)	23 (19–27)	26 (23–30)	0.330
MELD CHC/exception at LT (n = 29)^b^	25 (22–25)	25 (22–25)	25 (24–28)	22 (21–25)	0.281

HCC, hepatocellular carcinoma; LT, liver transplantation; MELD-Na, model for end-stage liver disease sodium; NASH, non-alcoholic steato-hepatitis; PBC, primary biliary cholangitis; PSC, primary sclerosing cholangitis. Bold values indicate statistically significant differences (p < 0.05).

a Some patients had mixed etiologies.

b One patient did not have a MELD score because the patient was assigned a 3F status on the LT waiting list, meaning the patient was in intensive care, not intubated, with a diagnosis of fulminant hepatitis. Data expressed as median (25th-75th percentiles) or as percentage. *P* values are reported for comparison between the three cohorts.

c Kruskall–Wallis test or Chi-square test.

Episodes of acute rejections (n = 12; 5 in cohort A, 2 in cohort B, and 5 in cohort C) and chronic rejection (n = 4; 3 in cohort A and 1 in cohort C) did not differ significantly between cohorts (*P* = 0.831 and *P* = 0.257, respectively) and had resolved by the time of data collection. Liver function markers (aspartate aminotransferase [AST], alanine aminotransferase [ALT], and alkaline phosphatase) were within normal ranges across cohorts, with no significant differences (data not shown).

### Comorbidities of Patients at the Time of LT and at 1-, 2-, and 3-years Post-transplant

**Figure 2:**
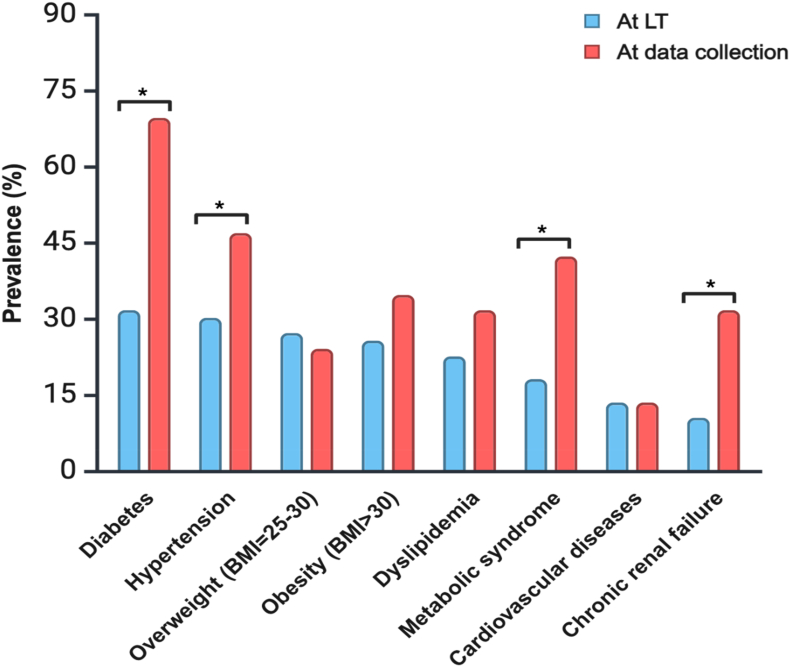
Comparison of comorbidities for all participants at transplantation and data collection (n = 66). Data are expressed as percentage. *P*-values are reported for the comparison of comorbidity percentages at transplantation and data collection for the three combined cohorts using McNemar's test. ∗: *P* value < 0.05). BMI, body mass index; LT, liver transplantation; NODAT, new onset diabetes after transplant.

At the time of LT, the most common comorbidities were type 2 diabetes (39.4%), followed by hypertension (30.4%), overweight (27.3%), obesity (25.8%), dyslipidemia (22.7%), metabolic syndrome (18.2%), cardiovascular disease (13.6%), and chronic kidney disease (10.6%). Post-LT, hypertension became the most prevalent (47.0%), followed by metabolic syndrome (42.4%), type 2 diabetes and obesity (34.8% each), dyslipidemia and chronic kidney disease (31.8% each), overweight (24.2%), and cardiovascular disease (13.6%) (Figure 2). NODAT occurred in 34.8% of patients, raising post-LT diabetes prevalence to 69.7% vs. 39.4% at LT (*P* < 0.001). Metabolic syndrome, hypertension, and chronic kidney disease also increased significantly post-LT (*P* < 0.001, *P* = 0.006, and *P* < 0.001, respectively) (Figure 2).

Table 2 presents the changes in comorbidities stratified by cohort, from the time of LT to the time of data collection after LT. While cohorts A (1-year post-LT) and cohort B (2-years post-LT) showed no significant differences, cohort C (3-years post-LT) had a significant rise in metabolic syndrome (14.8%–51.9%, *P* = 0.002) and chronic kidney disease (11.1%–40.7%, *P* = 0.021).

**Table 2:** Comorbidities at Transplantation and Data Collection for Each Cohort at 1-, 2-, and 3-Years Post-transplant (n = 66).

	Cohort A at LT n = 25	Cohort A at data collection n = 25	*P*-value^a^	Cohort B at LT n = 14	Cohort B at data collection n = 14	*P*-value^a^	Cohort C at LT n = 27	Cohort C at data collection n = 27	*P*-value^a^
Comorbidities, n (%)									
Diabetes type 2	7 (28.6)	7 (28.0)	1.000	4 (28.6)	5 (35.7)	1.000	10 (37.0)	14 (51.9)	0.125
NODAT	–	10 (40.0)	–	–	6 (42.9)	–	–	7 (25.9)	–
Hypertension	6 (24.0)	10 (40.0)	0.125	4 (28.6)	6 (42.9)	0.500	10 (37.0)	15 (55.6)	0.125
Overweight (BMI = 25–30)	7 (28.0)	5 (20.0)	0.625	5 (35.7)	5 (35.7)	1.000	6 (22.2)	6 (22.2)	1.000
Obesity (BMI >30)	4 (16.0)	8 (32.0)	0.125	5 (35.7)	7 (50.0)	0.625	8 (29.6)	8 (29.6)	1.000
Dyslipidemia	4 (16.0)	6 (24.0)	0.500	4 (28.6)	6 (42.9)	0.500	7 (25.9)	9 (33.3)	0.625
Metabolic syndrome	5 (20.0)	8 (32.0)	0.250	3 (21.4)	6 (42.9)	0.250	**4 (14.8)**	**14 (51.9)**	**0.002**
Cardiovascular diseases	4 (16.0)	4 (16.0)	1.000	3 (21.4)	3 (21.4)	1.000	2 (7.4)	2 (7.4)	1.000
Chronic renal failure	3 (12.0)	5 (20.0)	0.500	1 (7.1)	5 (35.7)	0.125	**3 (11.1)**	**11 (40.7)**	**0.021**

BMI, body mass index; LT, liver transplantation; NODAT, new onset diabetes after transplant. Data are expressed as percentage. *P*-values are reported for the comparison of comorbidity percentages at transplantation and data collection for each cohort. Bold values indicate statistically significant differences (p < 0.05).

a McNemar's test.

### Screening for Malnutrition, Sarcopenia, and Frailty at 1-, 2-, and 3-years Post-transplant

**Figure 3:**
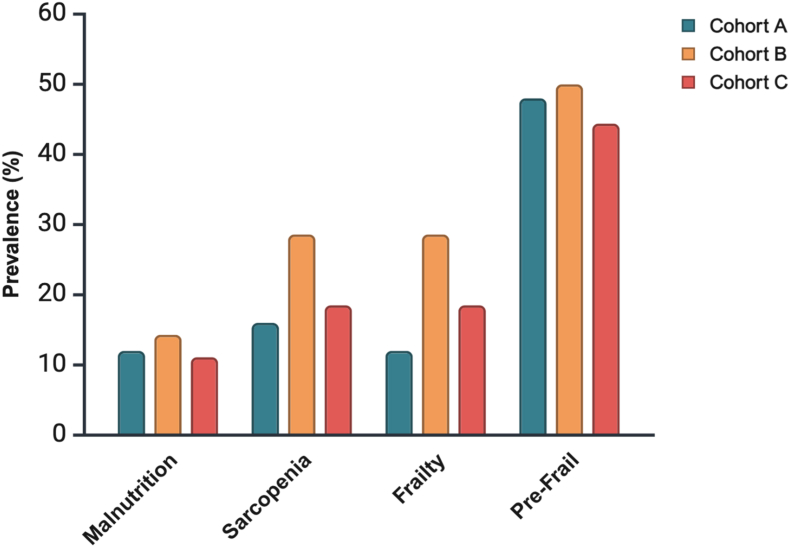
Prevalence of liver transplant recipients at risk of malnutrition, sarcopenia, and frailty at 1-, 2-, and 3-years post-transplant (n = 66). Cohort A = 1-year post-LT; Cohort B = 2 years post-LT; Cohort C = 3 years post-LT. Data are expressed as percentage. *P* values are reported for comparison among the three cohorts using the chi-square test. LT, liver transplantation.

The prevalence of patients at risk of malnutrition remained stable across the three cohorts (12.0%, 14.3%, and 11.1% for cohorts A, B, and C, respectively; *P* = 0.957). The prevalence of patients at risk of sarcopenia did not change significantly between cohorts, although the percentage was higher in cohort B (16.0%, 28.6%, and 18.5% for cohorts A, B, and C, respectively; *P* = 0.626). Regarding frailty, the prevalence of patients at risk was nearly the same as that for sarcopenia, with no significant difference between cohorts (12.0%, 28.6%, and 18.5% for cohorts A, B, and C, respectively; *P* = 0.436). Nearly half of the patients were considered prefrail in cohorts A, B, and C (48.0%, 50.0%, and 44.4%, respectively; *P* = 0.936) (Figure 3).

### Muscle Function of Liver Transplant Recipients

**Figure 4:**
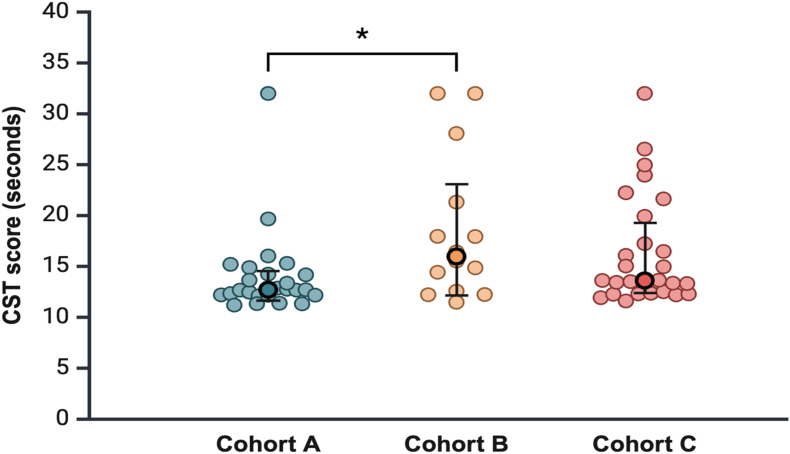
Muscle function of liver transplant recipients at 1-, 2-, and 3-years post-transplant (n = 66). Cohort A = 1-year post-LT; Cohort B = 2 years post-LT; Cohort C = 3 years post-LT. Data are expressed as the median (25th-75th percentiles) for each cohort. ∗: *P* value < 0.05 for comparison between cohort A and B using the Mann–Whitney U tests. LT, liver transplantation.

The median CST score was significantly higher in cohort B than in cohort A (16.0 s vs. 12.7 s, respectively; *P* = 0.046), while cohort C had a similar score to that of cohort A (13.6 s vs. 12.7 s, respectively; *P* = 0.145) (Figure 4). Four patients had a CST score of 32 s (n = 1 in cohort A, n = 2 in cohort B, and n = 1 in cohort C), indicating they were unable to complete the test.

### Physical Activity of Liver Transplant Recipients at 1-, 2-, and 3-years Post-transplant

Physical activity, assessed using the IPAQ, is presented in Table 3. Cohort A (1-year post-LT) performed VA–intensity activities for an average of 2 days per week for 15 min each, which is more frequent than cohorts B and C, who reported an average of no VA activities (*P* = 0.010). MA-intensity activity was similar across all three cohorts, with a median of 2 days per week for 30 min per day (*P* = 0.686). Walking was also similar across cohorts, with a median of 3 days per week for 60 min per day (*P* = 0.716). The median MET score was 1950.0 (824.0–4829.0) in cohort A, 783.8 (431.9–2561.3) in cohort B, and 834.0 (518.0–1794.0) in cohort C, with no significant differences between the cohorts (*P* = 0.096). Low physical activity was observed in 24.0%, 28.6%, and 40.7% of patients in cohort A, B, and C, respectively. Patients spent a median of 6 h being sedentary during the day, with no significant difference across cohorts (*P* = 0.411).

**Table 3:** Physical Activity of Liver Transplant Recipients at 1-, 2-, and 3-Years Post-transplant (n = 66).

	Total n = 66	Cohort A 1-year post-LT n = 25	Cohort B 2 years post-LT n = 14	Cohort C 3 years post-LT n = 27	*P*-value^a^
Vigorous-intensity activities (days per week)	**0 (0**–**2.3)**	**2 (0–3)**	**0 (0**–**0.5)**	**0 (0**–**0)**	**0.010**
Vigorous-intensity activities (minutes per day)	**0 (0**–**26.3)**	**15 (0–60)**	**0 (0–5)**	**0 (0**–**0)**	**0.011**
Moderate-intensity activities (days per week)	2 (1–5)	3 (0.5–6)	2 (1–6.3)	2 (0–4)	0.686
Moderate-intensity activities (minutes per day)	30 (9–60)	30 (3–60)	30 (18.8–67.5)	30 (0–60)	0.903
Walking (days per week)	3 (2–7)	4 (2–7)	3 (2.8–5)	3 (2–7)	0.716
Walking (minutes per day)	30 (15–60)	30 (20–60)	27.5 (13.8–32.5)	30 (15–60)	0.816
Total activity time (MET per week)	1112.3 (576.0–2874.0)	1950.0 (824.0–4829.0)	783.8 (431.9–2561.3)	834.0 (518.0–1794.0)	0.096
Level of activity (Low/moderate/high), n (%)	21/29/16 (31.8, 43.9, 24.2)	6/10/9 (24.0, 40.0, 36.0)	4/6/4 (28.6, 42.9, 28.6)	11/13/3 (40.7, 48.1, 11.1)	0.302
Sedentary time (hours per day)	6 (4–8)	5 (3.5–8)	6 (5–8)	6 (4–8)	0.411

LT, liver transplantation; MET, metabolic equivalent. Data are expressed as median (25th-75th percentiles) or as percentage. *P* values are reported for comparison among the three cohorts. Bold values indicate statistically significant differences (p < 0.05).

a Kruskall–Wallis test.

### Quality of Life and Employment Status of Liver Transplant Recipients at 1-, 2-, and 3-years Post-transplant

Among the nine sub-scores of the SF-36, the emotional well-being score was significantly higher at cohort A (80.0/100) compared to cohort C (64.0/100) (*P* = 0.039). Similarly, the health change score was significantly higher in cohort A (100/100) than both cohorts B (50/100) and C (50/100) (*P* < 0.001). All other sub-scores remained unchanged across the cohorts. The total physical health score and mental health score showed no significant differences between the cohorts (*P* = 0.557 and *P* = 0.682; respectively) (Table 4).

**Table 4:** Quality of Life of Liver Transplant Recipients at 1-, 2-, and 3-Years Post-transplant (n = 66).

	Total n = 66	Cohort A 1-year post-LT n = 25	Cohort B 2 years post-LT n = 14	Cohort C 3 years post-LT n = 27	*P* value^a^
Physical functioning	75.0 (53.8–90.0)	85.0 (65.0–92.5)	72.5 (43.8–90.0)	75.0 (50.0–85.0)	0.086
Role limitations due to physical health	25.0 (25.0–75.0)	50.0 (25.0–62.5)	25.0 (25.0–75.0)	25.0 (25.0–75.0)	0.488
Role limitations due to emotional problems	100 (58.3–100)	100 (16.7–100)	83.3 (58.3–100)	100 (66.7–100)	0.944
Social functioning	87.5 (62.5–100)	87.5 (62.5–100)	81.3 (59.4–100)	87.5 (62.5–100)	0.926
Pain	90.0 (42.5–100)	90.0 (45.0–100)	72.5 (35.0–100)	90.0 (32.5–100)	0.780
Energy/fatigue	50.0 (40.0–70.0)	55.0 (40.0–75.0)	50.0 (28.8–67.5)	50.0 (35.0–65.0)	0.604
Emotional well-being	**68.0 (56.0**–**81.0)**	**80.0 (66.0**–**88.0)**	**70.0 (51.0**–**92.0)**	**64.0 (52.0**–**72.0)**	**0.044**
General health	62.5 (35.0–81.3)	65.0 (40.0–85.0)	50.0 (35.0–76.3)	70.0 (30.0–85.0)	0.529
Health change	**75.0 (50.0**–**100)**	**100 (100**–**100)**	**50.0 (50.0**–**75.0)**	**50.0 (50.0**–**75.0)**	<**0.001**
Physical health score	61.3 (45.2–82.8)	65.0 (49.7–83.1)	53.4 (40.5–69.4)	67.5 (36.9–85.0)	0.557
Mental health score	72.1 (48.8–83.5)	71.5 (48.6–88.8)	74.5 (46.6–84.8)	68.8 (51.9–81.5)	0.682

LT, liver transplantation. Data are expressed as median (25th-75th percentiles). *P* values are reported for comparison among the three cohorts. Bold values indicate statistically significant differences (p < 0.05).

a Kruskall–Wallis test.

**Figure 5:**
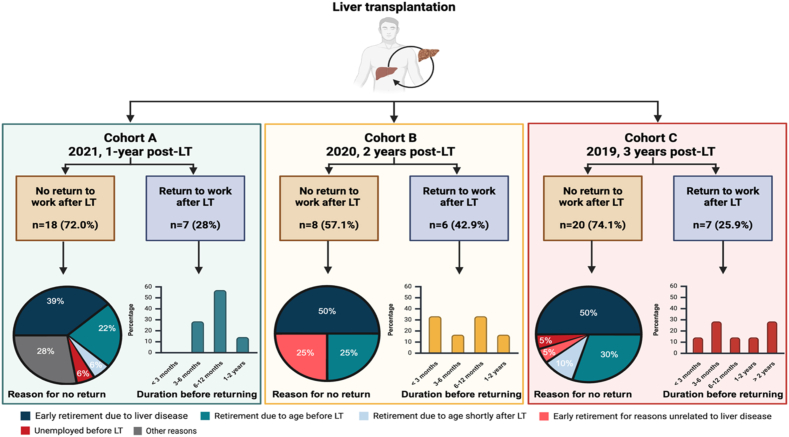
Employment status and work capacity of liver transplant recipients at 1-, 2-, and 3-years post-transplant (n = 66). Other reasons include relocation, work-related accidents, and a self-employed participant delaying return to work. LT, liver transplantation.

In cohort A, 72.0% of patients did not return to work, compared with 57.1% in cohort B and 74.1% in cohort C. The reasons for not returning to work are detailed in Figure 5 with the main reason being early retirement due to liver disease. Among those who resumed work, in cohort A, 28.6% did so within 6 months, 57.1% within 6–12 months, and 14.3% more than a year. In cohort B, 50.0% returned within 6 months, 33.3% between 6 and 12 months, and 16.7% after a year. In cohort C, 42.9% resumed work within 6 months, 14.3% between 6 and 12 months, and 42.9% after a year (Figure 5).

### Predictors of Long-term Risk of Malnutrition, Sarcopenia, and Frailty Post-transplant

Univariate logistic regressions were performed to assess factors associated with the risk of malnutrition, sarcopenia, and frailty (Table 5, Table 6, Table 7). These regressions included various potential risk factors such as age, sex, time since LT, comorbidities, medication, IPAQ score, CST score, and nutritional intake. Subsequently, multivariate logistic regressions were conducted, including all variables with a *P*-value <0.05.

**Table 5:** Univariate and Multivariate Logistic Regression for Risk of Malnutrition (n = 66).

	Univariate			Multivariate		
	OR	95% CI for OR	*P*-value	OR	95% CI for OR	*P*-value
**Age**	1.002	0.930–1080	0.952			
**Sex (male)**	0.217	0.025–1.886	0.166			
**Time since LT (days)**	1.000	0.998–1.002	0.847			
**Time on the waiting list, days**	0.999	0.996–1.001	0.358			
**Etiology of liver disease at LT, n (%)^+^**						
**NASH**	**7.235**	**1.325**–**39.497**	**0.022**			
**Alcohol**	0.790	0.266–5.704	0.790			
**Cholestatic (PBC/PSC)**	0.000	0.000–0.000	0.998			
**History of HCC at LT**	2.053	0.462–9.112	0.344			
**Child–Pugh at LT**	0.907	0.356–2.311	0.837			
**MELD-Na score at LT**	1.012	0.821–1.246	0.914			
**Comorbidities at data collection**						
Metabolic syndrome	5.200	0.986–27.409	0.052			
Hypertension	2.051	0.448–9.398	0.335			
Type 2 diabetes	2.937	0.637–13.538	0.167			
NODAT	0.587	0.109–3.175	0.536			
Obesity (BMI >30)	2.053	0.462–9.112	0.344			
Overweight (BMI = 25–30)	1.048	0.190–5.790	0.957			
Dyslipidemia	1.333	0.287–6.192	0.713			
Chronic renal failure	1.333	0.297–6.192	0.713			
Cardiovascular diseases	0.367	0.080–1.687	0.198			
**Imunosuppressants**						
Corticosteroids	0.271	0.031–2.363	0.238			
Tacrolimus	0.382	0.035–4.192	0.431			
Cyclosporine	2.619	0.239–28.753	0.431			
**IPAQ**	1.000	1.000–1.000	0.633			
**CST**	1.083	0.971–1.209	0.152			
**Sarcopenia risk**	**5.444**	**1.147**–**25.846**	**0.033**			
**Frailty risk**	**6.250**	**1.295**–**30.164**	**0.022**	**7.360**	**1.162**–**46.634**	**0.034**
**Prefrailty risk**	1.148	0.262–5.038	0.855			
**Calories (kcal/kg/d)**	**0.879**	**0.793**–**0.976**	**0.015**	**0.876**	**0.778**–**0.986**	**0.029**
**Carbohydrates (g/d)**	0.995	0.987–1.002	0.179			
**Protein (g/kg/d)**	0.145	0.017–1.273	0.082			
**Total lipids (g/d)**	**0.959**	**0.924**–**0.995**	**0.026**			

BMI, body mass index; CST, chair stand test; IPAQ, International Physical Activity Questionnaire; LT, liver transplantation; NASH, nonalcoholic steatohepatitis; PBC, primary biliary cholangitis; PSC, primary sclerosing cholangitis; MELD-Na, model for end-stage liver disease sodium; NODAT, new onset diabetes after transplant; OR, odds ratio. In the multivariate analysis, a forward selection model was used based on the likelihood ratio, including the variables that were significant in the univariate analysis. Bold values indicate statistically significant differences (p < 0.05).

**Table 6:** Univariate and Multivariate Logistic Regression for Risk of Sarcopenia (n = 66).

	Univariate			Multivariate		
	OR	95% CI for OR	*P*-value	OR	95% CI for OR	*P*-value
**Age**	1.018	0.953–1.089	0.594			
**Sex (male)**	0.457	0.112–1.859	0.274			
**Time since LT (days)**	1.000	0.998–1.002	0.863			
**Time on the waiting list, days**	0.999	0.996–1.001	0.236			
**Etiology of liver disease at LT**						
**NASH**	2.698	0.782–9.305	0.116			
**Alcohol**	1.982	0.575–6.837	0.279			
**Cholestatic (PBC/PSC)**	0.176	0.021–1.470	0.109			
**History of HCC at LT**	1.815	0.529–6.232	0.344			
**Child–Pugh at LT**	0.743	0.347–1.588	0.443			
**MELD-Na score at LT**	0.939	0.762–1.156	0.549			
**Comorbidities at data collection**	**0.142**	**0.034**–**0.582**	**0.007**			
Metabolic syndrome hypertension	0.960	0.284–3.240	0.948			
Type 2 diabetes	3.111	0.888–10.899	0.076			
NODAT	0.227	0.056–1.378	0.117			
Obesity (BMI >30)	2.698	0.782–9.305	0.116			
Overweight (BMI = 25–30)	0.923	0.220–3.872	0.913			
Dyslipidemia	2.171	0.626–7.530	0.222			
Chronic renal failure	1.445	0.409–5.105	0.567			
Cardiovascular diseases	4.267	0.957–19.030	0.057			
**Antidiabetic agents**						
Metformin	0.506	1.00–2.574	0.412			
Insulin	1.778	0.524–6.031	0.357			
Statins	2.388	0.684–8.332	0.172			
**Imunosuppressants**						
Corticosteroids	0.583	0.142–2.389	0.454			
Tacrolimus	0.216	0.027–1.701	0.146			
Cyclosporine	4.636	0.588–36.575	0.146			
**Vitamin D**	0.702	0.125–3.959	0.689			
**IPAQ**	0.039	0.998–1.000	0.999			
**CST**	**1.187**	**1.066**–**1.323**	**0.002**	**1.163**	**1.041**–**1.300**	**0.008**
**Malnutrition**	**5.444**	**1.147**–**25.846**	**0.033**			
**Frail**	**11.200**	**2.687**–**46.678**	<**0.001**			
**Prefrail**	0.649	0.188–2.244	0.495			
**Calories (kcal/kg/d)**	**0.919**	**0.856**–**0.988**	**0.021**	**0.924**	**0.855**–**1.000**	**0.049**
**Carbohydrates (g/d)**	0.995	0.989–1.001	0.117			
**Protein (g/kg/d)**	0.232	0.045–1.204	0.082			
**Total lipids (g/d)**	0.993	0.977–1.009	0.413			

BMI, body mass index; CST, chair stand test; IPAQ, International Physical Activity Questionnaire; LT, liver transplantation; NASH, nonalcoholic steatohepatitis; PBC, primary biliary cholangitis; PSC, primary sclerosing cholangitis; MELD-Na, model for end-stage liver disease sodium; NODAT, new onset diabetes after transplant; OR, odds ratio. In the multivariate analysis, a forward selection model was used based on the likelihood ratio, including the variables that were significant in the univariate analysis. Bold values indicate statistically significant differences (p < 0.05).

**Table 7:** Univariate and Multivariate Logistic Regression for Risk of Frailty (n = 66).

	Univariate			Multivariate		
	OR	95% CI for OR	*P*-value	OR	95% CI for OR	*P*-value
**Age**	1.008	0.945–1.076	0.800			
**Sex (male)**	1.316	0.367–4.715	0.673			
**Time since LT (days)**	1.000	0.999–1.002	0.623			
**Time on the waiting list, days**	1.000	0.999–1.002	0.521			
**Etiology of liver disease at LT**						
**NASH**	5.200	1.362–19.856	**0.016**			
**Alcohol**	1.555	0.431–5.610	0.500			
**Cholestatic (PBC/PSC)**	0.475	0.093–2.416	0.370			
**History of HCC at LT**	1.429	0.397–5.136	0.585			
**Child–Pugh at LT**	0.900	0.407–1.989	0.794			
**MELD-Na score at LT**	1.025	0.880–1.195	0.749			
**Comorbidities at data collection**	0.271	0.072–1.020	0.054			
Metabolic syndrome hypertension	1.750	0.493–6.213	0.387			
Type 2 diabetes	2.579	0.720–9.242	0.146			
NODAT	0.314	0.063–1.578	0.160			
Obesity (BMI >30)	3.325	0.917–12.052	0.067			
Overweight (BMI = 25–30)	1.051	0.247–4.472	0.946			
Dyslipidemia	**4.000**	**1.091**–**14.663**	**0.036**			
Chronic renal failure	1.696	0.468–6.149	0.421			
Cardiovascular diseases	**4.900**	**1.080**–**22.233**	**0.039**			
**Antidiabetic agents**						
Metformin	1.051	0.247–4.472	0.946			
Insulin	2.200	0.617–7.845	0.224			
Statins	2.857	0.791–10.326	0.109			
**Imunosuppressants**						
Corticosteroids	1.696	0.468–6.149	0.421			
Tacrolimus	0.192	0.024–1.529	0.119			
Cyclosporine	5.200	0.654–41.354	0.119			
**IPAQ**	0.999	0.999–1.000	0.071			
**CST**	**1.208**	**1.079**–**1.352**	**0.001**	**1.161**	**1.027**–**1.312**	**0.017**
**Malnutrition**	**6.250**	**1.295**–**30.164**	**0.022**			
**Sarcopenia**	**11.200**	**2.687**–**46.678**	<**0.001**	**5.761**	**1.182**–**28.071**	**0.030**
**Calories (kcal/kg/d)**	0.937	0.875–1.004	0.064			
**Carbohydrates (g/d)**	0.995	0.989–1.002	0.157			
**Protein (g/kg/d)**	0.198	0.035–1.128	0.068			
**Total lipids (g/d)**	0.984	0.964–1.004	0.124			

BMI, body mass index; CST, chair stand test; IPAQ, International Physical Activity Questionnaire; LT, liver transplantation; NASH, nonalcoholic steatohepatitis; PBC, primary biliary cholangitis; PSC, primary sclerosing cholangitis; MELD-Na, model for end-stage liver disease sodium; NODAT, new onset diabetes after transplant; OR, odds ratio.

In the multivariate analysis, a forward selection model was used based on the likelihood ratio, including the variables that were significant in the univariate analysis. Bold values indicate statistically significant differences (p < 0.05).

For the risk of malnutrition, the variables that were significant in the univariate analysis included having NASH as the underlying etiology of liver disease at LT, sarcopenia and frailty risks, total lipid and caloric intakes. In the multivariate analysis, only frailty risk and caloric intake remained significant, with an adjusted odds ratio (OR) of 7.360 (95% confidence interval [CI]: 1.162–46.634; *P* = 0.034) and an adjusted OR of 0.876 (95% CI: 0.778–0.986; *P* = 0.029), respectively (Table 5).

For the risk of sarcopenia, significant variables in the univariate analysis included the presence of metabolic syndrome, the CST score, risk of malnutrition and frailty, and caloric intake. Only the CST score and caloric intake remained significant in the multivariate analysis, with an adjusted OR of 1.163 (95% CI: 1.041–1.300; *P* = 0.008) and an adjusted OR of 0.924 (95% CI: 0.855–1.000; *P* = 0.049), respectively (Table 6).

Finally, for the risk of frailty, the variables that were significant in the univariate analysis included having NASH as the underlying etiology of liver disease at LT, the presence of dyslipidemia and cardiovascular disease at the moment of data collection, CST score, and risks of malnutrition and sarcopenia. Only the CST score and sarcopenia risk remained significant in the multivariate analysis, with an adjusted OR of 1.161 (95% CI: 1.027–1.312; *P* = 0.017) and an adjusted OR of 5.761 (95% CI: 1.182–28.071; *P* = 0.030), respectively (Table 7).

## DISCUSSION

### Malnutrition Risk in the Long Term After Liver Transplantation

Optimizing nutritional status after LT is essential for surgical success, rapid recovery, and long-term prognosis.^31^ Our study found that the prevalence of patients at risk of malnutrition remained stable at 1-, 2-, and 3-years post-LT (12.0%, 14.3% and 11.1%, respectively). The prevalence at 1-year post-LT aligns with that reported in the literature. Lim *et al.* identified rates of patients at risk of malnutrition at 1 year post-LT of 6.1%, 10.7%, and 10.7% using the Nutritional Risk Screening 2002, Malnutrition Universal Screening Tool, and Subjective Global Assessment, respectively.^32^ However, De Carvalho *et al.* found that 44% of LT recipients were classified with MA to severe malnutrition by the end of the first year using the protein-calorie malnutrition score.^33^ The high prevalence observed in the study by De Carvalho *et al.* may be explained by the use of a malnutrition assessment tool, making it more sensitive than screening tools.^33^

Caloric intake (kcal/kg/day) and frailty risk were the only significant predictors of malnutrition risk in our multivariable linear regression analysis. For each 1 kcal/kg/day increase in caloric intake, the risk of malnutrition decreased by 1.1-fold, whereas the presence of frailty increased the risk of malnutrition by 7.6-fold. Given the high prevalence of obesity and metabolic syndrome among LT recipients,^34^ nutritional strategies should focus on improving diet quality rather than simply increasing energy intake, to avoid excess caloric consumption and mitigate these risks.

Several physiological and metabolic changes can affect nutritional status and body composition following LT. The disruption of vagal innervation during surgery impairs the gut–liver–brain axis, potentially altering appetite regulation, nutrient metabolism, and overall energy homeostasis.^31^ After the first year, diet quality in LT recipients remains suboptimal, with notable deficiencies in specific micronutrients.^35^ Furthermore, immunosuppressive therapies, especially corticosteroids and calcineurin inhibitors, have a profound impact on protein metabolism.^3^ Emotional and behavioral factors, including dietary restrictions and psychological distress, may also influence eating behaviors and nutritional outcomes over the long term.^31^

### Sarcopenia and Frailty Risk in the Long Term After Liver Transplantation

Sarcopenia often worsens after LT, and patients who did not have it before LT may develop it after surgery.^35^^,^^36^ In our study, 16%, 28.6%, and 18.5% of patients were at risk of sarcopenia at 1-, 2- and, 3-years post-LT, respectively. Using computed tomography (CT) scans, Brown *et al.* reported a higher prevalence rates of sarcopenia (44%, 49%, and 43%) in patients at an average of 1-, 2-, and, 3-years post-LT.^37^ However, in Brown *et al.*‘s study, CT scans were performed for clinical care, which may have introduced a selection bias favoring the inclusion of sicker patients. Additionally, the study was retrospective, and the timing of CT scans varied across patients within the same cohort, limiting comparability.^37^ Tsien *et al.* reported a higher prevalence of sarcopenia assessed by CT scan, reaching 87% at 19 (±9) months post-LT.^38^ Frailty prevalence in our study was 12.0%, 28.6%, and 18.5% at 1-, 2-, and 3-years post-LT, respectively. Lai *et al.* reported a lower frailty prevalence of 7% at 1-year post-LT using the Liver Frailty Index, with only 40% of patients classified as physically robust.^39^

Muscle function was assessed using the CST in our study; we found that the median CST score was the highest at 2 years post-LT, indicating a further decrease in function (12.7 s, 16.0 s, and 13.6 s for 1-, 2-, and 3-years, respectively). A meta-analysis of older adults showed that times exceeding 11.4 s on the CST indicated worse-than-average performance than the general older adult population.^40^ In a multicenter study, a CST time exceeding 15 s was linked to nearly three-fold increase in mortality and an approximately two-fold increase in nonelective hospitalizations.^41^ The EWGSOP uses the same cutoff (>15 s) for the diagnosis of reduced muscle function in older adults.^5^ These findings indicate that our participants exceeded the average CST time for their age group across all three cohorts. Notably, in the 2 years post-LT cohort, CST times exceeded 15 s, a marker of reduced muscle function and elevated mortality and hospitalization risk. The reduction in muscle function in the 2-year cohort could be explained, at least in part, by the COVID-19 pandemic as these patients underwent LT in 2020, during lockdowns and reduced outdoor activity.

In our multivariable linear regression analysis, muscle function was a significant predictor of both sarcopenia and frailty, with each 1-s increase in CST time associated with a 1.2-fold higher risk for both sarcopenia and frailty. Caloric intake (kcal/kg/day) was also a significant predictor of sarcopenia, where each 1 kcal/kg/day increase was linked to a 1.1-fold higher risk. Additionally, sarcopenia significantly predicted frailty, with a 5.8-fold increased risk.

The persistence of sarcopenia and frailty risk beyond the first year after LT is intriguing. Advancing age may explain this observation; however, in the first year after LT, the yearly decline in skeletal muscle mass is greater in LT recipients than in the general elderly population, which suggests additional contributors.^37^ Immunosuppressive medications negatively impact muscle mass: calcineurin inhibitors upregulate slow-fiber-specific gene promoters and increase myostatin expression, thereby inhibiting muscle growth and regeneration; mammalian target of rapamycin (mTOR) inhibitors block muscle hypertrophy; and steroids cause type II muscle fiber atrophy.^37^ However, in our study, the use of immunosuppressive therapy was not associated with the risk of sarcopenia beyond one year after LT. Additional risk factors for sarcopenia following LT include physical inactivity, pre-existing sarcopenia, prolonged hospital stays, recurrent infections, biliary complications, insulin resistance, and renal dysfunction.^38^ Regarding renal dysfunction, our team previously found that post-LT Skeletal Muscle Index (SMI) was independently associated with pre-LT renal function markers, including glomerular filtration rate and serum creatinine.^36^ Another study using multivariate logistic regression identified age and chronic kidney disease progression as independent risk factors for sarcopenia in non-LT patients.^42^ Nevertheless, the mechanisms linking chronic kidney disease and sarcopenia after LT remain unclear, and the current evidence regarding the underlying pathways of post-LT sarcopenia warrants further investigation.

Previous studies reported that muscle loss before LT correlates with disease severity^43^ and is influenced by liver disease etiology (e.g. cholestatic and alcohol-related).^44^ In our study, however, liver disease severity (Child–Pugh and MELD scores) and the presence of alcohol-related or cholestatic liver disease at the time of LT were not significant predictors of post-transplant sarcopenia. In line with our findings, no studies to date have clearly demonstrated a link between liver disease severity or etiology and sarcopenia after LT.

### Physical Activity in the Long Term After Liver Transplantation

Increased physical activity post-LT improves quality of life, reduces metabolic and cardiovascular risks, reduces orthopedic complications, and enhances physical function.^31^ In our study, participants reported no VA physical activity, except at 1-year post-transplant, exercising on average of 2 days per week for 15 min. Similarly, Gitto *et al.* conducted a large multicenter study including 511 patients transplanted since 10.69 ± 7.11 years and found a median of no VA activity using the same questionnaire.^45^ The average MET score in our study was 1112.3 (576.0–2874.0), nearly half of the score reported by Gitto *et al.* Regarding sedentary behavior, our participants spent a median of 6^4^, ^5^, ^6^, ^7^, ^8^ hours per day sitting, approximately twice as much what reported by Gitto *et al.*.^45^ In 34 French-speaking Canadian adults aged 18 to 64, the reported median MET score was 3285 per day, nearly 3 times higher than that of LT recipient in our study.^46^ Our findings confirm that physical activity levels in LT recipients are low, with VA activity declining over time. There is an urgent need for a consensus on physical activity goals for LT recipients.

### Quality of Life and Employment Status in the Long Term After Liver Transplantation

Achieving adequate quality of life after LT is a key indicator of therapeutic success beyond survival.^9^ Our study found that quality of life remained stable across the three cohorts beyond 1 year, with an average physical health score of 61.3/100 and a mental health score of 72.1/100. The only changes observed were lower emotional well-being and health change scores at 3 years than 1-year post-LT. These findings align with previous reports showing that emotional well-being peaks within the first three months but declines over time.^47^ The decline in emotional well-being and health change scores may stem from early post-LT optimism and gratitude, which can lead to an overestimation of quality of life.^48^ As recovery progresses after LT, recipients face challenges in reintegration, independence, and work resumption.^48^ Long-term quality of life may be affected by immunosuppressive side effects, complications, residual effects of liver disease, and psychological distress, underscoring the need for ongoing support from transplant professionals.^49^ Additionally, post-transplant sarcopenia, frailty, and low physical activity significantly impact quality of life.^50^ The ability to resume employment is a key indicator of functional recovery and social reintegration after LT.^47^ In our study, only 28.0%, 42.9%, and 25.9% of participants in cohorts 1, 2, and 3, respectively, returned to work. These prevalences align with those reported in the literature, ranging from 26% to 57%, which remain lower than rates in the general or kidney transplant populations.^48^ Previous studies reported that most LT recipients who return to work do so within the first 6 months to 2 years after surgery.^49^ In our study, the majority (40%) resumed work within 6 months post-LT.

### Strengths and Limitations

The main strength of this study is that it is the first to report the prevalence of patients at risk of malnutrition, sarcopenia, and frailty in the long term after LT at specific time points. Additionally, all eligible patients who underwent LT between 2019 and 2021 at the CHUM were contacted, and data collection was conducted remotely. This approach ensures a more representative sample, avoiding bias toward patients living near the center, since transplant recipients were from across the province of Quebec. Finally, we used validated tools specifically designed for the French-Canadian population, as well as widely used tools, which facilitate comparisons with other studies. The SARC-F, SF-36, IPAQ, and OCDN questionnaires all have validated French versions, and the CST was administered orally in the patient's preferred language.

This study has certain limitations. First, the cross-sectional design limits the ability to establish causal relationships between parameters studied. Second, the absence of systematic data on malnutrition, sarcopenia, and frailty at the time of LT prevented direct pre- and post-transplant comparisons. Although limited nutritional information was occasionally available, records related to sarcopenia or frailty were virtually absent, restricting the interpretation of our findings. Third, the sample size of cohort B was relatively small due to the lower number of transplants performed in 2020, a consequence of the COVID-19 pandemic. Future studies with larger cohorts are recommended to confirm and validate our results. A potential strategy for improvement includes expanding recruitment across multiple LT centers, thereby increasing sample size and improving the generalizability of the findings. Additionally, involving patient partners could help refine recruitment materials and encourage participation in future studies. Finally, the FRAIL questionnaire has not been previously validated in French-speaking population.

We conclude that malnutrition, sarcopenia, and frailty risks remain prevalent up to 3 years post-LT. The percentage of patients at risk for these complications remains similar at 1-, 2-, and 3-years. Muscle function was notably reduced in the 2-year post-LT cohort was worse than that of age-matched older adults in all cohorts. About one-third of LT patients demonstrated low physical activity levels, with VA activity declining over time. Overall, the quality of life of LT patients remained stable except for emotional well-being and health changes, which were lower at 3-years compared with 1-year post-transplant.

These findings are concerning, and given the well-established links between malnutrition, sarcopenia, frailty, and morbidity and mortality post-LT, there is an urgent need for long-term follow-up in these patients. The first step involves an easy and simple screening for these complications for all LT recipients to identify those requiring comprehensive assessment. Nutritional and physical interventions should focus on optimizing body composition by reducing fat mass while increasing muscle mass and function. Multidisciplinary interventions are essential to optimize nutritional status, muscle health, and ultimately enhance the quality of life in LT recipients.

## Credit authorship contribution statement

Amal Trigui: Conceptualization; Data curation, Formal analysis; Investigation; Methodology; Visualization; Writing - original draft; Writing - review & editing. Crystèle Hogue: Visualization; Writing - review & editing. Mélanie Tremblay: Methodology; Visualization; Validation; Writing - review & editing. Geneviève Huard: Investigation; Writing - review & editing. Christopher F. Rose: Conceptualization; Supervision; Writing - review & editing. Chantal Bémeur: Conceptualization; Supervision; Visualization; Writing - review & editing; Funding acquisition.

## Funding

This research was supported by the discretionary funds of the principal investigator. Amal Trigui was supported by the Mission Universitaire de Tunisie en Amérique du Nord (MUTAN) doctoral research bursaries.

## Declaration of competing interest

The authors declare that they have no known competing interests or personal relationships that could have appeared to influence the work reported in this paper.
